# A phylogeny of Uniolinae with a new genus, *Eragrostiola* and combination, *E. spicata* (Poaceae, Chloridoideae, Eragrostideae, Uniolinae)

**DOI:** 10.3897/phytokeys.275.190083

**Published:** 2026-05-19

**Authors:** Paul M. Peterson, Konstantin Romaschenko, Robert J. Soreng, Yolanda Herrera Arrieta

**Affiliations:** 1 Department of Botany MRC-166, National Museum of Natural History, Smithsonian Institution, Washington, DC 20013-7012, USA National Museum of Natural History, Smithsonian Institution Washington, DC United States of America; 2 Instituto Politécnico Nacional, CIIDIR Unidad-Durango-COFAA, Durango, C.P. 34220, Mexico Instituto Politécnico Nacional, CIIDIR Unidad-Durango-COFAA Durango Mexico

**Keywords:** Classification, *
Eragrostis
*, ITS, phylogeny, plastid DNA sequences, Poaceae, *
Sporobolus
*, systematics, taxonomy

## Abstract

We present a molecular DNA phylogeny of *Uniola* utilizing three plastid (*rps16-trnK* spacer, *rps16* intron, *rpl32-trnL* spacer) and the nuclear ribosomal internal transcribed spacer (ITS) regions investigating 87 species of tribe Eragrostideae, including an increase in the sampling of species known to occur within *Uniola*. We describe a new genus, *Eragrostiola*, and make a new combination, *Eragrostiola spicata*, for a species that had been placed in *Eragrostis* for 134 years. *Uniola* and *Eragrostiola* include six species all native to the western hemisphere, share a common ancestor with the *Fingerhuthia* (*Entoplocamia* + *Tetrachne*) clade of African origin.

## Abstract (es)

Presentamos una filogenia del ADN molecular de *Uniola* utilizando tres regiones del cloroplasto (espaciador *rps16-trnK*, intrón *rps16*, espaciador *rpl32-trnL*) y las regiones espaciadoras transcritas internas ribosómicas nucleares (ITS) para 87 especies de la tribu Eragrostideae, incluido un aumento en el muestreo de especies de *Uniola*. Describimos un nuevo género, *Eragrostiola*, y creamos una nueva combinación, *Eragrostiola spicata*, para una especie que estaba clasificada en *Eragrostis* durante 134 años. *Uniola* y *Eragrostiola* incluyen a seis especies, todas ellas nativas del hemisferio occidental, que comparten un ancestro común *Fingerhuthia* (*Entoplocamia* + *Tetrachne*) de origen africano.

## Introduction

The genus *Uniola* L. includes five species restricted to the western hemisphere and is placed in the subtribe Uniolinae Clayton, along with three other genera, *Entoplocamia* Stapf, *Fingerhuthia* Nees ex Lehm., and *Tetrachne* Nees, all in the tribe Eragrostideae Stapf and subfamily Chloridoideae Kunth ex Beilschm. (Soreng et al. 2022; POWO 2025). *Uniola* is characterized in having ligules with a line of hairs, spikelets that are carinate (laterally flattened and compressed) with 3–9 (–13)-veined lemmas with midveins that have serrate or serrulate keels, lemma apices that are acute to obtuse (sometimes mucronate but never awned), glumes shorter than the spikelet, paleas with ciliate keels (sometimes winged), and caryopses with an embryo about half its length (Yates 2003; Clayton et al. 2016). *Uniola* historically included nine species (Hitchcock 1935, 1951) until Yates (1966a, 1966b, 1966c) recognized that five species, primarily from eastern North America with similar morphological characters, should be transferred to *Chasmanthium* Link, a genus now with seven species in the tribe Chasmanthieae W.V. Br. & B.N. Smith ex Sánchez-Ken & L.G. Clark, subfamily Panicoideae A. Braun (Davis and Soreng 1993; Sánchez-Ken and Clark 2003, 2010; Soreng et al. 2022). Additionally, Yates (1966a, 1966b) erected *Leptochloöpsis* Yates (most commonly treated as a synonym of *Uniola*, see Lægaard and Peterson 2001; Soreng et al. 2022) to include *Uniola condensata* Hitchc. and *U. virgata* (Poir.) Griseb., two species with smaller spikelets than those found in *U. paniculata* L. (type) and *U. pittieri* Hack. One additional species, *U. peruviana* Lægaard & Sánchez Vega, was described in Peru and is only known from the type (Lægaard and Sánchez Vega 1990).

While preparing a phylogeny of many species of *Eragrostis* Wolf (Peterson et al. in prep.) three accessions of *Eragrostis spicata* Vasey aligned outside of the *Eragrostis* clade. Here we present a new phylogenetic analysis using nuclear ITS and three plastid markers (*rpL32-trnL*, *rps16-trnK*, and *rps16*) with inclusion of these three accessions of *E. spicata* and an increase in the sampling of species known to occur within *Uniola*. It is interesting to note that Hackel (1909) described *Sporobolus tenuispica* Hack. from Paraguay that is currently a synonym of *E. spicata* (Peterson 2003; Peterson and Valdés-Reyna 2005; Peterson and Giraldo-Cañas 2012; POWO 2025). Therefore, the generic placement of this species requires further study. Based on our new molecular DNA-derived sequence analysis and interpretation of the morphological characters, we erect a new genus and make a new combination.

## Material and methods

### Taxon sampling

We sampled 101 individuals, representing 98 total species, eight species of Uniolinae, and four (4/6 = 67%) species of *Uniola*. A complete list of taxa including authorities, voucher information, and GenBank numbers is presented in Appendix 1.

We designed our study to characterize relationships among species of *Uniola* and within Uniolinae (*Entoplocamia*, *Fingerhuthia*, *Tetrachne*, and *Uniola*). We also included species from the Cotteinae Reeder (*Cottea pappophoroides* Kunth, *Enneapogon persicus* Boiss., *Kaokochloa nigrirostris* De Winter, and *Schmidtia pappophoroides* Steud. ex J.A. Schmidt), tribe Cynodonteae Dumort. [*Chloris barbata* Sw., *Cynodon plectostachyus* (K. Schum.) Pilg., *Eleusine indica* (L.) Gaertn., *Gymnopogon grandiflorus* Roseng., B.R.Arill. & Izag., *Halopyrum mucronatum* (L.) Stapf, *Kalinia obtusiflora* (E. Fourn.) H.L. Bell & Columbus, *Leptothrium rigidum* Kunth, *Oropetium capense* Stapf], Eragrostidinae J. Presl (75 species of *Eragrostis*), tribe Triraphideae P.M. Peterson (*Triraphis mollis* R.Br. and *T. ramosissima* Hack), and tribe Zoysieae Benth. [*Sporobolus pyramidatus* (Lam.) Hitchc. and *Zoysia macrantha* Desv.] (Soreng et al. 2022).

### Phylogenetic methods

All sequencing procedures of the plastid and ITS regions were performed in the Laboratory of Analytical Biology at the Smithsonian Institution. Detailed methods for DNA extraction, amplification, and sequencing are given in Romaschenko et al. (2012) and Peterson et al. (2010a, 2010b, 2012, 2014, 2015a, 2015b, 2016). Geneious Prime v.2020.1.4 (Kearse et al. 2012) was utilized for contig assembly of bidirectional sequences of *rps16-trnK* spacer, *rps16* intron, *rpl32-trnL* spacer, and ITS regions; and Muscle (Edgar 2004) to align consensus sequences and adjust the final alignment. The Bayesian trees were constructed with MrBayes v3.2.7 (Huelsenbeck and Ronquist 2001; Ronquist et al. 2012).

The evolutionary model parameters for each region were estimated with GARLI 2.0 (Zwickl 2006) (Table 1) and used as priors in Bayesian analysis. The combined data set was split into four partitions containing the ITS, *rpL32-trnL*, *rps16-trnK*, and *rps16* intron sequences. Bayesian analysis was initiated with random starting trees and was run for eight million generations with every 1000^th^ iteration being sampled. Upon completion of the search, the variance of split sequences was less than 0.01 and the potential scale reduction factor was close or equal to 1.0 indicating convergence of the chains (Huelsenbeck and Ronquist 2001). The effective sample size (ESS) value was greater than 100, and 25% of the sampled values were discarded. All compatible branches were saved. Posterior probabilities (PP) of ≥ 0.95 indicated a credible interval of probability.

**Table 1.** Characteristics of the four regions, *rps16-trnK, rps16* intron, *rpl32-trnL*, ITS, and parameters used in Bayesian analyses indicated by Akaike Information Criterion (AIC).

	rps16-trnK	rps16 intron	rpL32-trnL	Combined plastid data	ITS	Overall
Total aligned characters	1020	1019	1062	3101	745	3846
Number of sequences (success)	85	58	99	242	100	342
	(84.2%)	(57.4%)	(98.0%)	(79.9%)	(99.0%)	(84.7%)
Number of new sequences (ratio)	3	1	4	8	4	12
	(3.6%)	(1.7%)	(4.1%)	(3.3%)	(4.0%)	(3.5%)
Likelihood score (-lnL)	4248.88	3400.02	4144.03		9588.97	
Number of substitution types	4	4	4	-	4	-
Model for among-sites rate variation	gamma	gamma	gamma	-	Invar+Gamma	-
Substitution rates	1.8959	1.2463	1.6557	-	0.9989	-
	3.3086	1.5517	2.6459		2.8820	
	1.0000	0.3508	1.0000		2.0060	
	1.8959	1.0729	1.6557		0.5502	
	3.3086	1.5517	2.6459		7.3418	
	1.0000	1.0000	1.0000		1.0000	
Character state frequencies	0.2884	0.3714	0.3440	-	0.2500	-
	0.1535	0.1337	0.1464		0.2500	
	0.1551	0.1856	0.1440		0.2500	
	0.4030	0.3093	0.3655		0.2500	
Proportion of invariable sites	0.327	0.3890	0.3430	-	0.2916	-
Substitution model	TPM3u+F+R3	TVM+F+G4	TPM3u+F+R2	-	SYM+I+G4	-
Gamma shape parameter (α)	2.4860	0.5791	2.0110	-	0.7642	-

The bootstrap analysis (Felsenstein 1985) was performed using program IQ-Tree 3.0.1 implementing standard nonparametric bootstrap (with 10000 bootstrap replicates) to assess branch supports (Nguyen et al. 2015). Bootstrap values (BS) of ≥ 95% were interpreted as strong support.

A taxon duplication approach was applied to three individuals of *Eragrostis spicata* for which incongruence of plastid and ITS data was detected (Pirie et al. 2008; Gillespie et al. 2010; Pelser et al. 2010; Soreng et al. 2010; Peterson et al. 2015a, 2016, 2020, 2021, 2025a, 2025b). Combining all congruent data provides better resolution of phylogenetic trees, strengthens support for the nodes, and maximizes the informativeness and explanatory power of the character data used in the analysis (Huelsenbeck and Cunningham 1996). The three individuals of *E. spicata* were assigned two entries in the matrices: one containing only ITS and one containing only plastid sequences. This technique allowed us to identify the placements of the incongruent ITS and plastid sequences in the context of the *Eragrostis* and *Uniola* phylogeny, and to hypothesize multiple origins and elucidate complex evolutionary histories.

## Results

### Phylogenetic analyses

Twelve sequences (two for *Eragrostis sennii* Chiov. and 10 for three individuals of *E. spicata*) are newly reported in GenBank and the remaining were previously published sequences (Appendix 1) generated for earlier studies (Peterson et al. 2010a, 2015a, 2016; Barrett et al. 2020). Fifteen-point five percent (62/404) of the sequences (ITS and plastid) in our data set are missing (Appendix 1). Total aligned characters for individual regions and other parameters are shown in Table 1.

### Phylogeny

**Figure 1.**
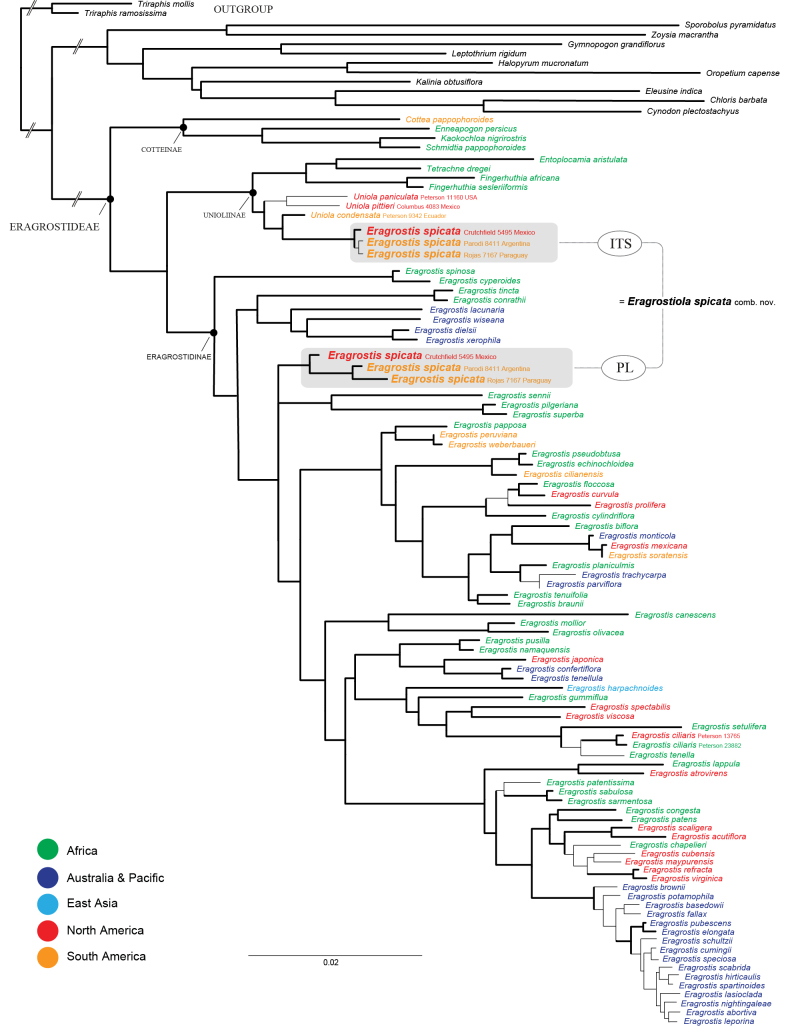
Reticulate origins of *Eragrostiola spicata*. Based on ITS sequences the species is found within *Uniola* and based on plastid markers (*rps16-trnK*, *rps16* intron, *rpl32-trnL*) it is found within *Eragrostis*. Geographic distribution (color); thick black branches in the phylogram indicate bootstrap of 95–100 and/or Bayes of 0.95–1.00. Scale bar: 2% substitutions per site.

The Bayesian tree based on plastid (*rps16-trnK* spacer, *rps16* intron, *rpl32-trnL* spacer) and ITS regions is well resolved and a monophyletic *Uniola* including three accessions of *Eragrostis spicata* (based on ITS sequences) is strongly supported (BS = 95–100; PP = 0.95–1.00) [Fig. 1]. Based on plastid sequences the three accessions of *E. spicata* form a strongly supported clade (BS = 95–100; PP = 0.95–1.00) which is depicted in a trichotomy deeply embedded within the *Eragrostis* clade. The first split within *Uniola* contains the *U. paniculata* + *U. pittieri* pair sister to a strongly supported clade (BS = 95–100; PP = 0.95–1.00) of *U. condensata* plus three individuals of *Eragrostis spicata*. Within the strongly supported Uniolinae (BS = 95–100; PP = 0.95–1.00) *Uniola* is sister to the *Fingerhuthia* (*Entoplocamia* + *Tetrachne*) clade.

### Taxonomy

#### Eragrostiola

Taxon classificationPlantaePoalesPoaceae

P.M.Peterson, Romasch. & Soreng gen. nov.

55571C14-347F-51FE-9A45-E51309A7AD16

urn:lsid:ipni.org:names:77380112-1

##### Type.

*Eragrostiola spicata* (Vasey) P.M. Peterson, Romasch. & Soreng, Bot. Gaz. 16(5): 146. 1891.

##### Description.

Caespitose ***perennials*** with innovations, without rhizomes. **Culms** 75–100 cm tall, erect, glabrous. ***Sheaths*** hirtellous on the margins when immature, apices glabrous or hairy, the hairs shorter than 0.5 mm long; ***ligules*** 0.2–0.3 mm long, a line of hairs; ***blades*** 20–40 cm long, 2–5 (–6) mm wide, flat to involute, glabrous abaxially, scabrous adaxially. ***Inflorescence*** a panicle 22–40 cm long, 0.3–0.6 cm wide, narrow, spikelike, densely-flowered; ***primary branches*** shorter than 1.2 cm long, closely appressed, spikelet-bearing to the base; ***pulvini*** glabrous; ***pedicels*** 0.1–0.6 mm long, mostly appressed, hirtellous. ***Spikelets*** 1.4–2.2 mm long, 0.9–1.2 mm wide, ovate, strongly laterally flattened (carinate), stramineous to light greenish, with (1–) 2–3 florets; ***disarticulation*** basipetal, in the rachilla below the individual florets or at the base of the florets, glumes persistent; ***glumes*** elliptic to ovate, 1-veined, hyaline, keels ciliolate; ***lower glumes*** 0.7–1 mm long; ***upper glumes*** 0.9–1.3 mm long, apices obtuse; ***lemmas*** 1.5–2.1 mm long, 3-veined, ovate, membranous to hyaline, midveins serrulate to ciliate often greenish, apices acute to obtuse; ***paleas*** 1.1–1.6 mm long, hyaline, not wider than the lemmas, 2-keeled, distally minutely winged, keels ciliolate, apices obtuse. ***Flowers*** perfect; ***lodicules*** 2; ***stamens*** 3; ***anthers*** 2, 0.3–0.4 mm long, reddish-brown. ***Caryopses*** 0.7–1 mm long, ellipsoid, somewhat ventrally flattened, smooth to faintly striate, reddish-brown. 2*n* = 40 (Brown 1950).

##### Etymology.

The name is derived from the genus *Eragrostis* combined with ‘ola’ referring to *Uniola*, therefore alluding to being intermediate between these two genera.

##### Species.

Monotypic with a single taxon, *Eragrostiola spicata*.

##### Distribution and habitat.

It is native from southern Texas to Mexico, and in Paraguay and Argentina. This species grows in wet meadows, pastures, and prairies usually in deep sandy clay loam soils or saline substrates in low-lying areas, 0–500 m.

#### Eragrostiola spicata

Taxon classificationPlantaePoalesPoaceae

(Vasey) P.M.Peterson, Romasch. & Soreng comb. nov.

F8C9B42D-664C-5E86-9D9F-525E1E518ACF

urn:lsid:ipni.org:names:77380113-1

Eragrostis spicata Vasey, Bot. Gaz. 16(5): 146. 1891. Type: Mexico, Baja California Sur, San José del Cabo, 12 Sep 1890, *T.S. Brandegee 10* (holotype: US-1761638!).
= Sporobolus tenuispica Hack., Repert. Spec. Nov. Regni Veg. 6(21–26): 344. 1909. Type: Paraguay, Gran Chaco, Boquerón, in campis altis in regione cursus inferiores fluminis Pilcomayo, Jun 1906, *T. Rojas 258* (holotype: W19160026618 (image!); isotypes: BAA00002845 (image!) col. Typus 2931 fragm. ex hb. Hassler, MVFA0000660 (image!), US-2891485! fragm. ex hb. Hassler).

## Discussion

It is not surprising that the generic placement of what we are now calling *Eragrostiola spicata* was interpreted differently by two prominent agrostologists, including Vasey (1891) describing it in *Eragrostis* and Hackel (1909) describing it in *Sporobolus*. This species indeed includes characteristics suggesting alignment in *Sporobolus* in having narrow, spike-like panicles and a line of hairs for a ligule. *Eragrostiola spicata* was included in *Eragrostis* for 134 years since individuals have (1–) 2 or 3-flowered spikelets and prominently 3-veined lemmas. In addition to the above-mentioned characteristics, *Eragrostiola* includes salient features of *Uniola* in having strongly laterally flattened, i.e., carinate spikelets, lemmas with midveins that have serrate or serrulate keels, acute to obtuse lemma apices (sometimes mucronate but never awned), and paleas with distally minutely winged keels.

In our ITS-based phylogeny *Eragrostiola spicata* is sister to *U. condensata* and embedded within *Uniola*, whereas based with plastid markers it aligns within *Eragrostis*. This incongruence is common among chloridoid grasses and can be used to develop evolutionary hypotheses (Peterson et al. 2015a, 2015b, 2016, 2021, 2025a, 2025b; Barrett et al. 2020). The plastid lineage of *Eragrostiola spicata* may have been donated through a hybridization event involving an unknown ancient lineage of *Eragrostis* since it forms a trichotomy deeply embedded in our phylogeny (Fig. 1), whereas the ITS signal clearly aligns as sister to *U. condensata* embedded within *Uniola* and sharing those same morphological characteristics mentioned in the previous paragraph.

Our first impression was to recognize this fully fertile species, which appears to be a product of an ancient hybridization event, within *Uniola* since it shares many unifying morphological features. However, *Eragrostiola* does have 3-veined lemmas with hyaline glumes (only *U. virgata* includes lemmas that are 3–5-veined), whereas all other species within *Uniola* have 5–9 (–13)-veined lemmas and glumes that are membranous, chartaceous or coriaceous. Future molecular DNA sequence studies including samples of *U. virgata* and *U. peruviana* are needed to elucidate the phylogeny of *Uniola*, and the possible resurrection of *Leptochloöpsis* (now a synonym of *Uniola*), which has many morphological traits not found in *Uniola* s.s. (Yates 1966b).

*Uniola* and *Eragrostiola* include six species native to the western hemisphere, whereas the other three genera (*Entoplocamia*, *Fingerhuthia*, and *Tetrachne*) include a total of four species native to Africa (Soreng et al. 2022; POWO 2025). *Uniola* and *Eragrostiola* share a common ancestor most likely of African origin with the *Fingerhuthia* (*Entoplocamia* + *Tetrachne*) clade (see Fig. 1), and the subtribe most likely arose in Africa 13.84 mya (crown age) [Gallaher et al. 2022].

## Supplementary Material

XML Treatment for Eragrostiola

XML Treatment for Eragrostiola spicata

## Appendix 1

**Table A1.** Taxon voucher (collector, number, and where the specimen is housed), country of origin, and GenBank accession for DNA sequences of *rps16–trnK*, *rps16 intron*, *rpl32–trnL*, and ITS regions; bold indicates new accession; a dash (–) indicates missing data; * indicates GenBank data.

	Taxa	Voucher	Country	*rps16–trnK*	*rps16 intron*	*rpl32–trnL*	ITS
1	*Chloris barbata* Sw.	Peterson 22255& Saarela (US)	Mexico, Sinaloa	GU360514	GU360435	GU359873	GU359320
2	*Cottea pappophoroides* Kunth	Peterson 21463, Soreng, LaTorre & Rojas Fox (US)	Peru, Ancash	GU360600	GU360456	GU359842	GU359237
3	*Cynodon plectostachyus* (K. Schum.) Pilg.	Troupin 11610 (MO)	Rwanda, Mutara	GU360592	GU360449	GU359890	GU359247
4	*Eleusine indica* (L.) Gaetrn.	Peterson 21362, Saarela & Flores Villegas (US)	Mexico, Mexico	GU360496	GU360472	GU359797	GU359338
5	*Enneapogon persicus* Boiss.	Peterson 24265, Soreng, Romaschenko & Mbago (US)	Tanzania, Shinyanga	PV982173	PV982205	PV959311	PV954963
6	*Entoplocamia aristulata* (Hack. & Rendle) Stapf	Seydel 187 (US)	Namibia	GU360492	GU360468	GU359793	GU359342
7	*Eragrostis abortiva* (R. Br.) Steud.	Wannan 2216 (MEL)	Australia, Queensland	MK872799	MK872654	MK872566	MK863241
8	*Eragrostis acutiflora* (Kunth) Nees	Peterson 8627 (US)	Panama, Bocas Del Toro	**–**	–	MK872416	MK863092
9	*Eragrostis atrovirens* (Desf.) Trin. ex Steud.	Swallen 5268 (US)	USA, Florida	–	–	MK872423	MK863098
10	*Eragrostis basedowii* Jedwabn.	Forster 20782 & Holland (MEL)	Australia, Queensland	MK872673	MK872581	–	MK863101
11	*Eragrostis biflora* Hack.	Smook 6546 (US)	South Africa, Orange Free State	MK872674	–	MK872426	MK863102
12	*Eragrostis braunii* Schweinf.	Brunt 1304 (US)	Kenya, Nakuru	MK872676	–	MK872428	MK863104
13	*Eragrostis brownii* (Kunth) Nees	Peterson 14459, Soreng & Rosenberg (US)	Australia, Western Australia	MK872677	–	MK872429	MK863105
14	*Eragrostis canescens* C.E. Hubb.	Wiehe 202 (US)	Malawi, Nyasaland	MK872679	–	MK872431	MK863107
15	*Eragrostis chapelieri* (Kunth) Nees	Peterson 23890, Soreng, Romaschenko & Abeid (US)	Tanzania, Ruvuma	MK872681	MK872582	MK872433	MK863109
16	*Eragrostis cilianensis* (All.) Vignolo ex Janch.	Peterson 21461, Soreng, Torre & Rojas Fox (US)	Peru, Ancash	MK872682	–	MK872434	MK863110
17	*Eragrostis ciliaris* (L.) R. Br.	Peterson 13765 (US)	Mexico, Jalisco	MK872683	–	MK872435	MK863111
18	*Eragrostis ciliaris* (L.) R. Br.	Peterson 23882, Soreng, Romaschenko & Abeid (US)	Tanzania, Mtwara	MK872684	MK872583	MK872436	MK863112
19	*Eragrostis confertiflora* (J.M. Black) J.M. Black	Thompson 730 & Simon (MO)	Australia, Queensland	MK872685	MK872584	MK872437	MK863113
20	*Eragrostis congesta* Oliv.	Peterson 24077, Soreng, Romaschenko & Abeid (US)	Tanzania, Rukwa	MK872686	MK872585	MK872438	MK863114
21	*Eragrostis conrathii* (Hack.) S.M. Phillips	Schweickerdt 1652 (US)	South Africa, Pretoria	–	–	MK872569	MK863244
22	*Eragrostis cubensis* Hitchc.	Hitchcock 23359 (US)	Cuba	–	–	MK872441	MK863117
23	*Eragrostis cumingii* Steud.	Saarela 1733, Peterson, Soreng & Judziewicz (US)	Australia, Northern Territory	MK872689	MK872587	MK872444	MK863120
24	*Eragrostis curvula* (Schrad.) Nees	Peterson 21308, Saarela & Flores Villegas (US)	Mexico, Mexico	MK872690	–	MK872448	MK863124
25	*Eragrostis cylindriflora* Hochst.	Kolberg 1068 & Tholkes (US)	Namibia, Khomas	MK872691	–	MK872449	MK863125
26	*Eragrostis cyperoides* (Thunb.) P. Beauv.	Davidse 33267 (MO)	South Africa, Cape	MK872656	MK872570	MK872402	MK863080
27	*Eragrostis dielsii* Pilg.	Peterson 14399, Soreng & Rosenberg (US)	Australia, Western Australia	GU360537	GU360400	GU359779	GU359297
28	*Eragrostis echinochloidea* Stapf	Klaassen 1847, Rugheimer, Mannheimer, Hochobes & Hasheela (US)	Namibia, Karas	MK872697	–	MK872455	MK863131
29	*Eragrostis elongata* (Willd.) J. Jacq.	Peterson 14364, Soreng & Rosenberg (US)	Australia, Western Australia	MK872699	–	MK872457	MK863133
30	*Eragrostis fallax* Lazarides	Saarela 1897, Peterson, Soreng & Judziewicz (US)	Australia, Northern Territory	MK872707	MK872597	MK872465	MK863141
31	*Eragrostis floccosa* Hack. ex Jedwabn.	Schweickerdt 1689 (US)	South Africa, Transvaal	MK872711	–	MK872469	MK863145
32	*Eragrostis gummiflua* Nees	Smook 7065 (US)	South Africa, Transvaal	MK872714	–	MK872472	MK863148
33	*Eragrostis harpachnoides* Hack.	Soreng 5288, Peterson & Sun Hang (US)	China, Yunnan	GU360611	GU360382	GU359815	GU359113
34	*Eragrostis hirticaulis* Lazarides	Michell 3802 (DNA)	Australia, Northern Territory	MK872715	MK872601	MK872473	MK863149
35	*Eragrostis japonica* (Thunb.) Trin.	Peterson 9552 (US)	USA, Louisiana	–	–	MK872478	MK863154
36	*Eragrostis lacunaria* F. Muell.	Saarela 1641, Peterson, Soreng & Judziewicz (US)	Australia, Northern Territory	MK872721	MK872604	MK872481	MK863157
37	*Eragrostis lappula* Nees	Klaassen 1318 & Bortscsh, Hochobe, Kruger & Lutombi (US)	Namibia, Okavango	MK872727	–	MK872487	MK863163
38	*Eragrostis lasioclada* Merr.	Blake 8572 (US)	Australia, Queensland	–	–	MK872407	MK863084
39	*Eragrostis leporina* (R. Br.) R.L. Barrett & P.M. Peterson	Saarela 1803, Peterson, Soreng & Judziewicz (US)	Australia, Northern Territory	MK872663	MK872577	MK872412	MK863088
40	*Eragrostis maypurensis* (Kunth) Steud.	Jimenez 2208 (US)	Costa Rica	MK872730	MK872611	MK872491	MK863166
41	*Eragrostis mexicana* (Hornem.) Link	Peterson 24874 & Romaschenko (US)	Mexico, San Luis Potosí	MK872732	MK872612	MK872493	MK863168
42	*Eragrostis mollior* Pilg. ex R.E. Fr.	Peterson 24159, Soreng, Romaschenko & Abeid (US)	Tanzania, Iringa	MK872735	MK872614	MK872496	MK863170
43	*Eragrostis monticola* (Gaudich.) Hillebr.	Wood 2902 & Lau (US)	USA Hawaii, Maui	MK872736	MK872615	MK872497	MK863171
44	*Eragrostis namaquensis* Nees ex Schrad.	Peterson 23911, Soreng, Romaschenko & Abeid (US)	Tanzania, Ruvuma	MK872737	MK872616	MK872498	MK863172
45	*Eragrostis nightingaleae* R.L. Barrett & P.M. Peterson	Saarela 1750, Peterson, Soreng & Judziewicz (US)	Australia, Northern Territory	MK872742	MK872620	MK872502	MK863176
46	*Eragrostis olivacea* K. Schum.	McCallum-Webster 62 (US)	Kenya, Nairobi	MK872748	–	MK872508	MK863182
47	*Eragrostis papposa* (Roem. & Schult.) Steud.	Pappi 4119 (US)	Eritrea, Amasen	MK872750	–	MK872511	MK863185
48	*Eragrostis parviflora* (R.Br.) Trin.	Peterson 14445, Soreng & Rosenberg (US)	Australia, Western Australia	GU360506	GU360465	GU359804	GU359331
49	*Eragrostis patens* Oliv.	Peterson 23901, Soreng, Romaschenko & Abeid (US)	Tanzania, Ruvuma	MK872753	MK872627	MK872513	MK863187
50	*Eragrostis patentissima* Hack.	Oakes 508 (US)	South Africa, Orange Free State	MK872754	–	MK872514	MK863188
51	*Eragrostis peruviana* (Jacq.) Trin.	Dillon 3216, Molau & Matekaitis (US)	Peru, Arequipa	MK872756	–	MK872517	MK863191
52	*Eragrostis pilgeriana* Dinter ex Pilg.	Klaassen 1298, Batsch, Hochobes, Kruger & Lutombi (US)	Namibia, Okavango	MK872757	–	MK872518	MK863192
53	*Eragrostis planiculmis* Nees	Godfrey 1364 & Story (US)	South Africa, Cape of Good Hope	MK872758	–	MK872519	MK863193
54	*Eragrostis potamophila* Lazarides	Saarela 1898, Peterson, Soreng & Judziewicz (US)	Australia, Northern Territory	MK872759	MK872629	MK872521	MK863195
55	*Eragrostis prolifera* (Sw.) Steud.	Gould 12607 (US)	Mexico, Yucatan	–	–	MK872522	MK863196
56	*Eragrostis pseudobtusa* De Winter	Smook 6198 (US)	South Africa, Transvaal	–	–	MK872523	MK863197
57	*Eragrostis pubescens* (R. Br.) Steud.	Simon 4411 (US)	Australia, Queensland	MK872761	–	MK872525	MK863199
58	*Eragrostis pusilla* Hack.	Smook 5226 (US)	Namibia, Otjozondjupa	–	–	MK872526	MK863200
59	*Eragrostis refracta* (Muhl. ex Elliott) Scribn.	Rosen 4610 (US)	USA, Texas	MK872762	–	MK872527	MK863201
60	*Eragrostis sabulosa* (Steud.) Schweick.	Van Rensburg 2544 (US)	Zambia, Namwala	MK872764	–	MK872529	MK863203
61	*Eragrostis sarmentosa* (Thunb.) Trin.	Laegaard 15731 (US)	Zimbabwe, Cleveland Dam Park	MK872765	–	MK872530	MK863204
62	*Eragrostis scabrida* (C.E.Hubb.) R.L.Barrett	Lazarides 4772 (US)	Australia, Queensland	GU360497	GU360459	GU359799	GU359317
63	*Eragrostis scaligera* Salzm. ex Steud.	Harvey 8333 (US)	USA, Montana	–	–	MK872531	MK863205
64	*Eragrostis schultzii* Benth.	Saarela 1798, Peterson, Soreng & Judziewicz (US)	Australia, Northern Territory	MK872766	MK872632	MK872532	MK863206
65	*Eragrostis sennii* Chiov.	Gilbert 1369 & Thulin (US)	Kenya, Mandera	–	–	**PZ025869**	**PZ025869**
66	*Eragrostis setulifera* Pilg.	Renvoize 2339 & Abdallah (US)	Tanzania, Iringa	MK872774	–	MK872539	MK863214
67	*Eragrostis soratensis* Jedwabn.	Peterson 16274, Cano, LaTorre, Ramirez & Susanibar Cruz (US)	Peru, Ayacucho	GU360532	GU360395	GU359831	GU359287
68	*Eragrostis spartinoides* Steud.	Saarela 1843, Peterson, Soreng, & Judziewicz (US)	Australia, Northern Territory	–	MK872636	MK872540	MK863215
69	*Eragrostis speciosa* (Roem. & Schult.) Steud.	Saarela 1825, Peterson, Soreng & Judziewicz (US)	Australia, Northern Territory	MK872780	MK872641	MK872546	MK863221
70	*Eragrostis spectabilis* (Pursh) Steud.	Peterson 14240, Weakley & LeBlond (US)	USA, North Carolina	MK872781	–	MK872547	MK863222
71	*Eragrostis spicata* Vasey ≡ *Eragrostiola spicata* (Vasey) P.M. Peterson, Romasch. & Soreng	Crutchfield 5495 (US)	Mexico, Tamaulipas	**PZ036108**	–	**PZ036104**	**PZ025870**
72	*Eragrostis spicata* Vasey ≡ *Eragrostiola spicata* (Vasey) P.M. Peterson, Romasch. & Soreng	Parodi 8411 (US)	Argentina, Formosa	**PZ036109**	**PZ036107**	**PZ036105**	**PZ025871**
73	*Eragrostis spicata* Vasey ≡ *Eragrostiola spicata* (Vasey) P.M. Peterson, Romasch. & Soreng	Rojas 7167 (US)	Paraguay, Chaco Boreal	**PZ036110**	–	**PZ036106**	**PZ025872**
74	*Eragrostis spinosa* (L. f.) Trin.	Swallen 10469 (US)	South Africa, Northern Cape	–	–	MK872405	MK863083
75	*Eragrostis superba* Peyr.	Peterson 24203, Soreng, Romaschenko & Mbago (US)	Tanzania, Tanga	MK872782	MK872642	MK872548	MK863223
76	*Eragrostis tenella* (L.) P. Beauv. ex Roem. & Schult.	Peterson 24233, Soreng, Romaschenko & Mbago (US)	Tanzania, Tanga	MK872783	MK872643	MK872549	MK863224
77	*Eragrostis tenellula* (Kunth) Steud.	Saarela 1693, Peterson, Soreng & Judziewicz (US)	Australia, Northern Territory	MK872788	MK872647	MK872554	MK863229
78	*Eragrostis tincta* S.M. Phillips	Kemp 1290 (MO)	Swaziland	MK872800	MK872655	MK872567	MK863242
79	*Eragrostis trachycarpa* (Benth.) Domin	Coveny 16369 & Whalen (US)	Australia, New South Wales	MK872795	–	MK872561	MK863236
80	*Eragrostis virginica* (Zuccagni) Steud.	Strong 2964, Aronica, Simmons & Tice (US)	USA, Virginia	MK872796	–	MK872562	MK863237
81	*Eragrostis viscosa* (Retz.) Trin.	Gould 12761 (US)	Mexico, Chiapas	–	–	MK872563	MK863238
82	*Eragrostis weberbaueri* Pilg.	Peterson 21807 & Soreng (US)	Peru, Ancash	GU360530	GU360393	GU359829	GU359304
83	*Eragrostis wiseana* (C.A. Gardner & C.E. Hubb.) R.L. Barrett & P.M. Peterson	Peterson 14345, Soreng & Rosenberg (US)	Australia, Western Australia	GU360703	GU360288	GU359986	GU359137
84	*Eragrostis xerophila* Domin	Saarela 1624, Peterson, Soreng & Judziewicz (US)	Australia, Northern Territory	MK872798	MK872653	MK872565	MK863240
85	*Fingerhuthia africana* Nees ex Lehm.	Bester 7070 (MO)	South Africa, Northern Cape	PV982180	PV982211	PV959318	PV954969
86	*Fingerhuthia sesleriiformis* Nees	Phillipson 608 (MO)	South Africa, Eastern Cape	PV982181	PV982212	PV959319	PV954970
87	*Gymnopogon grandiflorus* Roseng., B.R. Arill. & Izag.	Peterson 16642 & Refulio-Rodriguez (US)	Peru, Apurimac	GU360581	GU360383	GU359816	GU359200
88	*Halopyrum mucronatum* (L.) Stapf	Peterson 23837, Soreng, Romaschenko & Abeid (US)	Tanzania, Lindi	KX582970	KX582903	KX582650	KX582374
89	*Kalinia obtusiflora* (E. Fourn.) H.L. Bell & Columbus	Reeder 5530 & Reeder (US)	USA, Arizona	KX582975	KX582908	KX582658	KX582382
90	*Kaokochloa nigrirostris* De Winter	Smook 7779 (MO)	Namibia, Kaokoveld	–	–	PV959320	PV954971
91	*Leptothrium rigidum* Kunth	Davidse 3281 (MO)	Jamaica, Kingston Parish	KF827794	KF827727	KF827662	KF827541
92	*Oropetium capense* Stapf	Venter 9939 & Venter (MO)	South Africa, Free State	KM011120	KM010917	KM010692	KM010324
93	*Schmidtia pappophoroides* Steud. ex J.A. Schmidt	Peterson 24182, Soreng, Romaschenko & Abeid (US)	Tanzania, Dodoma	PV982184	PV982215	PV959323	PV954974
94	*Sporobolus pyramidatus* (Lam.) Hitchc.	Garcia 3757 (CIIDIR)	Mexico, Durango	KM011263	KM011064	KM010860	KM010476
95	*Tetrachne dregei* Nees	Jarman 120 (US)	South Africa, Free State	GU360622	GU360365	GU359904	GU359218
96	*Triraphis mollis* R. Br.	Peterson 14344, Soreng & Rosenberg (US)	Australia, Western Australia	GU360669	GU360336	GU359933	GU359187
97	*Triraphis ramosissima* Hack.	Seydel 4278 (US)	Namibia	GU360651	GU360338	GU359931	GU359188
98	*Uniola condensata* Hitchc.	Peterson 9342 & Judziewicz (US)	Ecuador, Chimborazo	GU360649	GU360340	GU359927	GU359191
99	*Uniola paniculata* L.	Peterson 11160, Annable & Valdes-Reyna (US)	USA, Texas	GU360648	GU360341	GU359926	GU359192
100	*Uniola pittieri* Hack.	Columbus 4083 (RSA)	Mexico, Jalisco	–	AY509526*	–	–
101	*Zoysia macrantha* subsp. *walshii* M.E. Nightingale	Loch 435 (US)	Australia	GU360642	GU360345	GU359922	GU359197

## Additional information

### Artificial Intelligence (AI) use

The authors accept full responsibility for the content of the manuscript, including the disclosure of any use of AI.

No AI tools were used in the preparation of this manuscript.

### Author ORCIDs

P.M. Peterson https://orcid.org/0000-0001-9405-5528

K. Romaschenko https://orcid.org/0000-0002-7248-4193

R.J. Soreng https://orcid.org/0000-0002-8358-4915

Y. Herrera Arrieta https://orcid.org/0000-0003-3814-6518

### Data availability

All of the data that support the findings of this study are available in the main text.
